# The intimate relationship between gut microbiota and cancer immunotherapy

**DOI:** 10.1080/19490976.2018.1527167

**Published:** 2018-10-19

**Authors:** Arielle Elkrief, Lisa Derosa, Laurence Zitvogel, Guido Kroemer, Bertrand Routy

**Affiliations:** a Centre de recherche du Centre hospitalier de l’Université de Montréal (CRCHUM), Montréal, Qc, Canada; b Division of Internal Medicine Department of Medicine, McGill University, Montreal, Qc, Canada; c Department of Oncology, Gustave Roussy Cancer Campus (GRCC), Villejuif, France; d Department of Oncology, Institut National de la Santé Et de la Recherche Médicale (INSERM) U1015, Villejuif, France; e Department of Oncology, Équipe Labellisée, Ligue Nationale contre le Cancer, Villejuif, France; f Department of Oncology, Univ. Paris-Sud, Université Paris-Saclay, Gustave Roussy, Villejuif, France; g Metabolomics and Cell Biology Platforms, GRCC, Villejuif, France; h Sorbonne Paris Cité, Paris Descartes University, Paris, France; i Equipe 11 Labellisée—Ligue Nationale contre le Cancer, Centre de Recherche des Cordeliers, Paris, France; j Department of Oncology, Institut National de la Santé et de la Recherche Médicale, Paris, France; k Department of Oncology, Pierre et Marie Curie University, Paris, France; l Department of Oncology, Pôle de Biologie, Hôpital Européen Georges Pompidou, Assistance Publique—Hôpitaux de Paris, Paris, France; m Department of Women’s and Children’s Health, Karolinska University Hospital, Stockholm, Sweden; n Hematology-Oncology Division, Department of Medicine, Centre Hospitalier de l’Université de Montréal (CHUM), Montréal, Qc, Canada

**Keywords:** Gut microbiota, cancer, oncology, immunotherapy, fecal transplantation, profiling gut microbiome, host pathogen interactions

## Abstract

Immunotherapy is widely used to treat a large variety of malignancies and has revolutionized the therapeutic approach to cancer. Major efforts are ongoing to identify biomarkers that predict response to immunotherapy as well as new strategies to improve ICI efficacy and clinical outcomes. Studies have shown that the gut microbiome determines the extent to which ICIs may invigorate the anticancer immune response. Here, the authors review recent studies that have described the effects of the gut microbiota on the efficacy of CTLA-4 and PD-1 inhibitors and outline potential future clinical directions of these findings.

## Immunotherapy: moving away from directly targeting tumor cells

Immunotherapy is widely used to treat a large variety of malignancies and has revolutionized the therapeutic approach to cancer. Immune check-point inhibitors (ICI) have been successful in the treatment of both solid cancers (including melanoma and renal cell carcinoma, RCC, non-small cell lung cancer, NSCLC, or mismatch-repair deficient colorectal cancer) and hematological malignancies. ICIs function by suppressing the interaction of T-lymphocyte inhibitory receptors with their ligands on malignant or myeloid cells, thereby re-stimulating the T-lymphocyte-mediated immune response against tumor-associated antigens.^1^

The ICIs approved in clinical practice are monoclonal antibodies (mAbs) that inhibit the interaction between programmed cell death protein 1 (PD-1) and its ligand PD-L1 ^1^ In addition, mAbs targeting cytotoxic T-lymphocyte antigen 4 (CTLA-4) can enable cytotoxic lymphocytes to attack tumor cells.^1^ For example, treatment with pembrolizumab, a PD-1 inhibitor, was associated with significantly longer progression-free and overall survival and with fewer adverse events than was with standard-of-care chemotherapy in patients with advanced NSCLC, provided that more than 50% of the tumor cells expressed PDL.-1^2^ More recently, the combination of nivolumab (a PD-1 inhibitor) and ipilimumab (a CTLA-4 inhibitor) was associated with significantly longer progression-free compared with first line standard-of-care chemotherapy in advanced NSCLC patients.^3^ Contrary to the previous study, the response was not influenced by the level of PD-L1 expression within the tumor. Widely used in the metastatic setting, ICIs are now also being used in both the neoadjuvant and adjuvant settings for the treatment of non-metastatic cancers. A recent pilot study in early stage NSCLC showed that neoadjuvant nivolumab induced a pathological response in 45% of resected tumors without significant adverse events or a delay in the surgical procedure.^4^ Different from standard chemotherapy, ICI mobilized the immune system to not only target the tumor cells but also to cause a long-term memory T cell response that is likely to decrease the risk of residual or recurrent disease.^5^

Unfortunately, despite these unprecedented results there is still an unmet clinical need for the majority of patients across all cancer types. As a result, major efforts are ongoing to identify biomarkers that predict response to ICI as well as new strategies to improve ICI efficacy and clinical outcomes. Potential mechanisms of action of resistance to immunotherapy with ICI include low mutational burden, low levels of antigens in the tumor cells^6^, lack of potential immunogenic pretreatment with radiation or chemotherapy^7^, as well as local or systemic immunosuppression.^8^ These considerations have led to the development of both tumor and immune-related biomarkers. However, these markers have not been shown to be consistently sensitive or specific. For example, patients bearing PD-L1 negative tumors can respond to anti-PD-L1 therapy, and a considerable fraction of patients with PD-L1 positive cancers are non-responders.^9^

Indeed, the immunity of each individual patient at baseline or in response to ICI is influenced by a complex and interconnected set of factors including host, tumor, and environmental variables.^5^ These factors determine the threshold of the anti-cancer immune response also known as the cancer immune set-point.^10^ Recent studies highlighted that the gut microbiome acts as an imperative determinant of this cancer immune set-point.^11^ In other words, the gut microbiome may determine the extent to which ICIs may invigorate the anticancer immune response.^12^

## The gut microbiome and efficacy of CTLA-4 and PD-1 inhibitors

In 2015, two studies published in *Science* unraveled this association between the gastrointestinal microbiota and ICI efficacy in preclinical mouse models, showing that CTLA-4 and PD-1 blockade only reduced tumor growth in mice that carry within their commensal intestinal flora *Bacteroides* and *Bifidobacterium* species, respectively.^13,14^ Thus, tumors in broad-spectrum antibiotic (ATB) treated mice or in germ-free (GF) mice that lacked *Bacteroides* species were resistant to the CTLA-4 blockade. Response to CTLA-4 inhibition was regained by oral gavage of the GF mice with *Bacteroides fragilis*.^14^ This intervention induced colonization of *B. fragilis* in the mouse gut flora and consequently caused T-cell helper (TH1) responses to increase in the lymph nodes closest to the tumor, thereby improving the efficacy of the CTLA-4 blockade. Finally, transplantation of *Bacteroides* species-rich feces into GF mice induced significant response to CTLA-4 blockade.^14^

Chaput et al confirmed the importance of specific commensals in both clinical response and toxicity. In a cohort of 26 patients with metastatic melanoma treated with ipilimumab, patients whose baseline microbiota was *Faecalibacterium* rich has longer PFS and OS compared to those whose microbiota was *Firmicutes* poor. However, the patients enriched with these commensals also had more frequent occurrence of ipilimumab-induced colitis.^15^

We recently confirmed the key role of the gut microbiota in determining the clinical activity of ICI-based therapies in cancer patients.^16^ First, we studied the impact of antibiotics (ATB) among 249 NSCLC, renal cell cancer (RCC) and urothelial cancer patients treated with ICI.^16^ Sixty-nine (28%) of these patients received ATB in a window period of 60 days before or 30 days after the first injection of ICI. The ATB were prescribed most commonly for dental, urinary or pulmonary infections, and only a few patients required hospitalization. The most common ATB prescribed were B-lactams, fluoroquinolones and macrolides. The baseline characteristics between the ATB-treated and ATB-free groups were similar. However, similarly to the mouse model, we found that patients in the ATB-group was affected by significantly lower overall survival. Analyzing each tumor type separately, overall survival or progression-free-survival were significantly shorter in the ATB-treated group. Both univariate and multivariate Cox-regression models indicated that ATB use constitutes an independent marker of non-response to ICI in NSCLC and RCC patients. To validate the clinical relevance of this observation, we recently analyzed two new cohorts of 239 NSCLC and 121 RCC patients treated with ICI. We observed that following one course of ATB the microbiota recovered to approximately 80% within one month.^17^ For this reason, we focused our attention on patients receiving ATB within the 30 days before starting ICI. When taking into consideration this treatment window, both progression-free and overall survival were shorter in the ATB-group for both cohorts.^16^ Altogether, these results suggest that, in current medical practice, modifications of the microbiota have a major impact on the outcome of cancer immunotherapy.

Using the quantitative metagenomics platform at the Institut National de la Recherche Agronomique (INRA), the gut microbiome was characterized in patients with NSCLC and RCC prior to PD-1 blockade. These analyses led to the hypothesis that the intestinal microbiota can help to classify patients receiving ICI in responders (Rs) and non-responders (NRs) defined by standardized radiologic criteria RECIST 1.1 criteria. Specific bacterial species such as *Akkermanasia muciniphila* and *Alistipes indistinctus* were present in a disproportionately large number in the feces from Rs compared to NRs, using best clinical response (stable disease or partial response compared to progression) as the clinical criterion for the distinction between Rs and NRs. *A. muciniphila* was significantly associated with favorable clinical prognosis in 100 NSCLC and RCC patients (p = 0.004). *A. muciniphila* was also more abundant among feces from patients with progression-free survival (PFS) longer than 3 months (p = 0.028). When analyzing the gut microbiota composition in a validation cohort of NSCLC (n = 27) and RCC (n = 26) patients, we established that *A. muciniphila* was more abundant in patients with PFS longer than 3 months compared to patients with PFS shorter than 3 months.^16^

Gopalakrishnan et al. confirmed the importance of the gut microbiota for the immunotherapy of patients with metastatic melanoma patients. This group from MD Anderson used 16 S RNA sequencing technology on feces from 43 melanoma patients to demonstrate that *Fecalibacterium spp*. were overrepresented in responder patients.^18^ Further confirming that the gut microbiome composition influences clinical response in melanoma patients, Matson et al. showed that *Bifidobacterium longum and adolescentis, Collinesella aerofaciens, and Parabacteiodes merdae* were more abundant in the stools from R patients as compared to NRs, in which *Ruminococcus obeum and Roseburia intestinalis* were more abundant.^19^

## Specific commensals and their role in immune response

We studied memory T-cell responses stimulated by PD-1 blockade to explore the association between the gut microbiota and the immune system. The response of CD4^+^ and CD8^+^ T cells harvested from the peripheral blood from PD-1 treated NSCLC (n = 27) and RCC (n = 28) patients to specific bacteria was associated with favorable clinical outcome. Higher IFNγ production by CD4^+^ and CD8^+^ T cells in response to *A. muciniphila* correlated with prolonged progression-free survival. In contrast, no association was found between clinical outcome and memory T-cell responses against 10 randomly selected commensals.^16^

To confirm that PD-1 efficacy and resultant clinical responses were truly determined by the dominance of certain bacterial species, we performed the gavage of feces harvested at diagnosis from NSCLC patients (four Rs and four NRs) into “avatar mice”, thereby recolonizing these initially ATB-treated (sterilized) mice with patient-derived microbiota. These mice were then inoculated with mouse MCA-205 fibrosarcoma cells. Once tumors were established, the mice were treated with PD-1 blockade. It was demonstrated that mice transplanted with stool from R NSCLC patients were sensitive to PD-1 blockade.^16^ In contrast, mice transplanted with stool from NR NSCLC patients were resistant to the treatment. These findings were reproduced transplantation of stool samples from seven RCC patients into ATB-treated mice inoculated with luciferase-expressing renal cell cancer.^16^

Similar experiments using patients’ feces were performed by Gajewski and Wargo groups and these results validated that patient’s gut microbiota transplanted in murine model dictated the response to ICI and influenced systemic immune tone. Indeed, FMT from R feces promoted upregulation of T cell CD8^+^, while downregulating immunosuppressive (Treg) in the tumors.^13^

In order to demonstrate the clinical potential of manipulating the gut microbiota to increase the response to ICI, we orally supplemented mice that received FMT from NR with *A. mucinphila* and other commensals enriched in R patients including *Alistipes indistinctus* and *Enterococcus hirae* which successfully restored responsiveness to PD-1 blockade against the MCA-205 tumor model.^16^ In these models, oral gavage with *A. muciniphila*, led to an increase of IL-12 production by dendritic cells, and a shift of T central memory trafficking from the mesenteric lymph nodes towards the tumor microenvironment. In addition, microbiota manipulation with immunogenic commensals significantly decreased the Treg compartments within the tumor.^16^

## Future directions

The gut microbiome influences the efficacy of both PD-1 and CTLA-4 blockades as well as important clinical outcomes. However, many questions remain unanswered. First, efforts must be undertaken in order to understand the shift in the composition of the gut microbiota that results from exposure to ATB. For example, the mechanisms of immunomodulation by *A. muciniphila* and other commensals need to be further described.^20^ Moreover, the clinical significance of the gut microbiota as a novel biomarker of ICI response must be validated in prospective studies.

Milestones that remain to be achieved include the standardization of the methods of feces sample collections, as well as the extraction and sequencing of bacterial DNA. In addition, culturomics analysis must continue to be performed in order to unveil new bacteria species, with special attention given to fastidious commensals.

**Figure 1.**
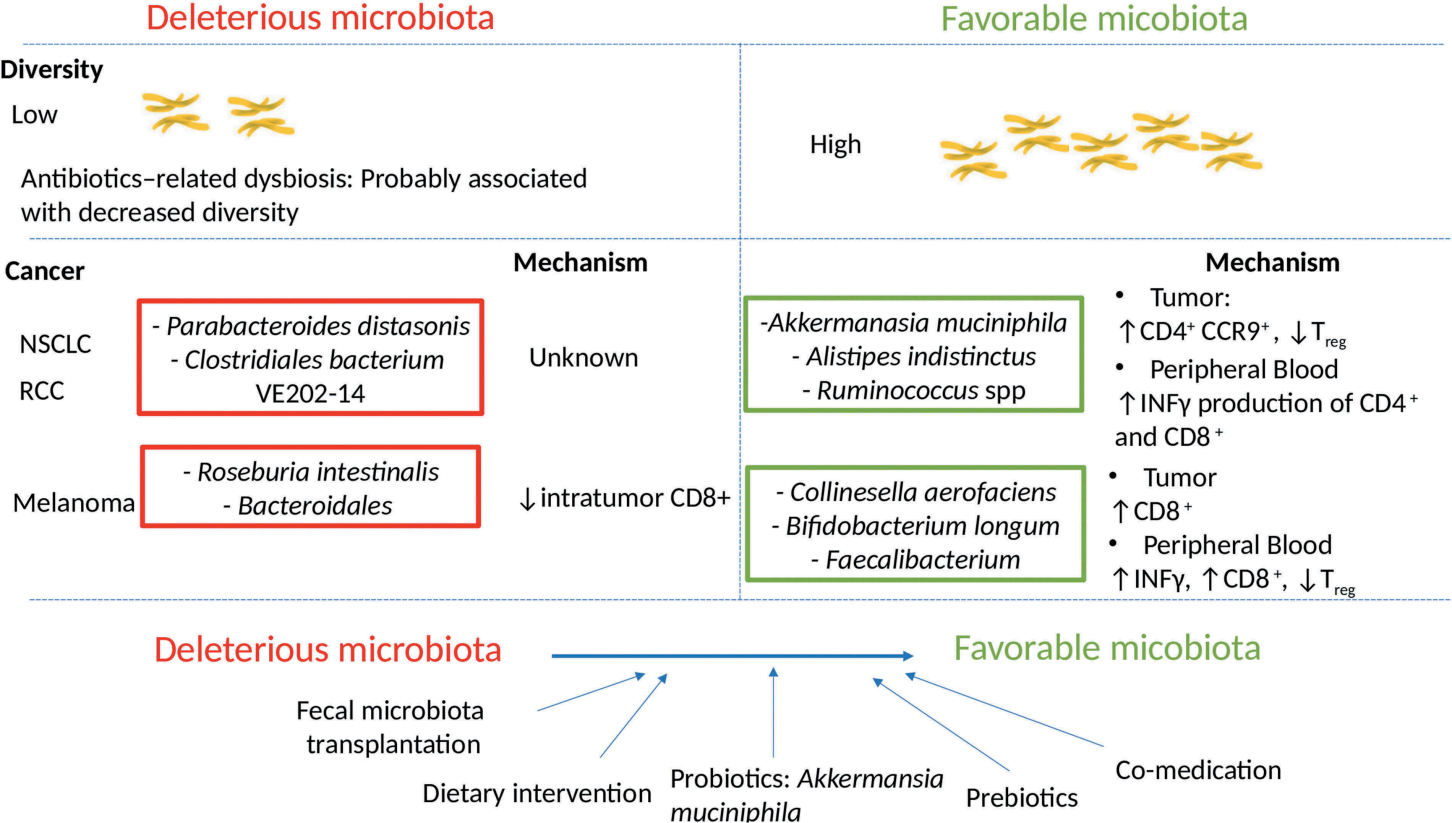
Link between microbiota diversity and composition with immune checkpoint inhibitors clinical response. Upper panel: Patients that did not respond to ICI have a lower microbiota diversity. Prescription of antibiotics (in favour of a lower microbiota diversity) prior to commence ICI was associated with a poorer clinical response.Middle panel: Bacteria overrepresented in deleterious microbiota associated with non-responders, and bacteria enriched in responders with favourable microbiota and their respective mechanisms influencing the anti-cancer immune responseLower panel: Strategies to manipulate the gut microbiota and transform an unfavourable to favourable microbiota to enhance ICI response.

The manipulation of the gut microbiota in an effort to improve clinical outcomes represents a second paradigm shift in oncology (Figure 1). Several methods are currently being studied including the use of prebiotics, probiotics, fecal microbiota transplantation or capsule loaded with bacteria and these efforts merit further study. Additional experimentations are required to understand the biology, the pharmacokinetics, and optimal delivery and duration of these potential interventions in order to improve outcomes of patients living with cancer.
